# Post-colostomy internal hernia of the stomach treated with laparoscopic gastropexy: a case report

**DOI:** 10.1186/s40792-022-01455-0

**Published:** 2022-05-30

**Authors:** Yoichiro Tada, Junpei Orihara, Yamato Wada, Ei Uchinaka, Tomohiro Osaki, Keigo Ashida, Shigeru Tatebe, Seika Kuroda, Yasuaki Hirooka

**Affiliations:** Division of Surgery, Tottori Prefectural Central Hospital, 730 Ezu, Tottori City, Tottori 680-0901 Japan

**Keywords:** Internal hernia, Colostomy, Laparoscopic gastropexy

## Abstract

**Background:**

Internal hernias are formed by the protrusion of internal organs through an aperture formed congenitally or postoperatively. Internal hernias are most commonly associated with the small intestine. Only two cases of a post-sigmoid colostomy internal hernia of the stomach have been reported. This hernia arises from the space between the lifted sigmoid colon and the left abdominal wall. In the two aforementioned cases, treatment comprised suturing of the sigmoid colon to the lateral abdominal wall and changing of the intraperitoneal route to an extraperitoneal one. Herein, we present a very rare case who underwent laparoscopic gastropexy for a post-sigmoid colostomy internal hernia of the stomach.

**Case presentation:**

Our patient, a 67-year-old woman, was undergoing chemoradiation for rectal cancer and planned to undergo abdominoperineal resection. However, tumor perforation resulted in a high fever and a right gluteal abscess; therefore, a sigmoid colostomy was performed through the intraperitoneal route in the left lower abdomen. One month after the surgery, the patient presented to our emergency room with vomiting, abdominal pain, and abdominal distension. Computed tomography revealed a markedly distended stomach caused by the obstruction of the pylorus secondary to the colostomy; laparoscopic gastropexy was performed subsequently and the postoperative course was uneventful.

**Conclusions:**

This is the first report on the laparoscopic gastropexy treatment of a post-sigmoid colostomy internal hernia of the stomach; our findings may help physicians manage such hernias.

## Background

**Fig. 1:**
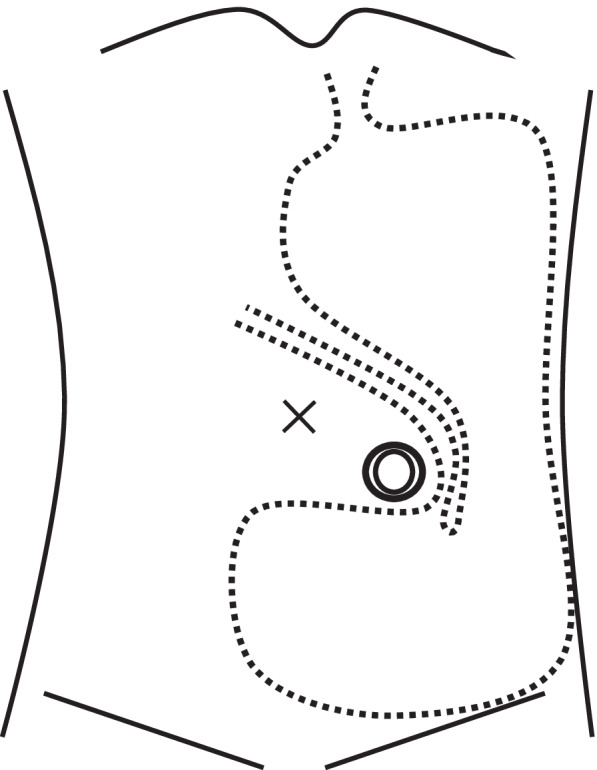
The stomach dilates after eating and descends into the pelvis through the space between the lifted sigmoid colon and the lateral abdominal wall. The antrum is obstructed by the lifted sigmoid colon, resulting in the internal hernia of the stomach. A dotted line and a double circle indicate the stomach and the sigmoid colostomy, respectively

An internal hernia is a protrusion of the internal organs through an aperture formed congenitally or postoperatively [1]. Internal hernias are most commonly associated with the small intestine and are rarely associated with the stomach. Internal hernias of the stomach comprise parastomal hernias [2] and post-colostomy internal hernias [3, 4]. The stomach dilates after eating and descends into the pelvis through the space between the lifted sigmoid colon and the left abdominal wall. Therefore, the antrum is obstructed by the sigmoid colon, resulting in the internal hernia (Fig. 1).

Notably, only two cases of post-colostomy internal hernias have been reported. The first case was treated by suturing the sigmoid colon to the lateral abdominal wall [3], while the second case was treated by changing the intraperitoneal route to an extraperitoneal one [4]. Both treatments are for closing an aperture, and the use of gastropexy for treatment has not been reported yet.

Herein, we present a very rare case of a 67-year-old patient who underwent laparoscopic gastropexy [5] for an internal hernia of the stomach that developed after a sigmoid colostomy.

## Case presentation

A 67-year-old woman was undergoing chemoradiation therapy for lower rectal cancer which arose from the right side of the anal canal and invaded the anal sphincter muscle. She was scheduled to undergo a laparoscopic or robot-assisted abdominoperineal resection. However, tumor perforation resulted in a high fever and a right gluteal abscess; therefore, a sigmoid colostomy was performed through the intraperitoneal route in the left lower abdomen.

**Fig. 2:**
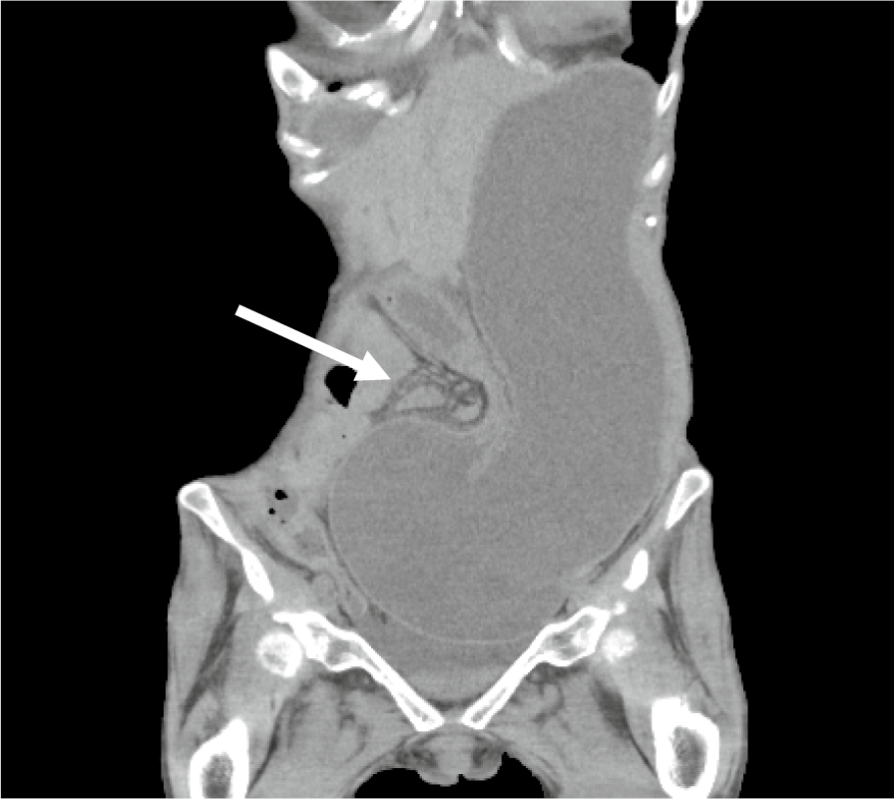
CT showed the stomach was markedly dilated due to the obstruction near the pylorus caused by the lifted sigmoid colon. A white arrow points to the sigmoid colostomy

One month after the surgery, the patient presented to our emergency room with vomiting, abdominal pain, and abdominal distension. Computed tomography (CT) revealed marked dilation of the stomach due to the obstruction of the pylorus caused by the sigmoid colostomy (Fig. 2). After insertion of a nasogastric tube and drainage of approximately 2000 mL of gastric juice, the abdominal symptoms improved rapidly. Because laparoscopic abdominoperineal resection was scheduled for 2 months later and preoperative gastroscopy showed no lesions that could cause stenosis, we decided to observe her without surgery. However, she developed an recurrent internal hernia twice; thus, we performed a laparoscopic gastropexy [6] for the internal hernia of the stomach.

**Fig. 3:**
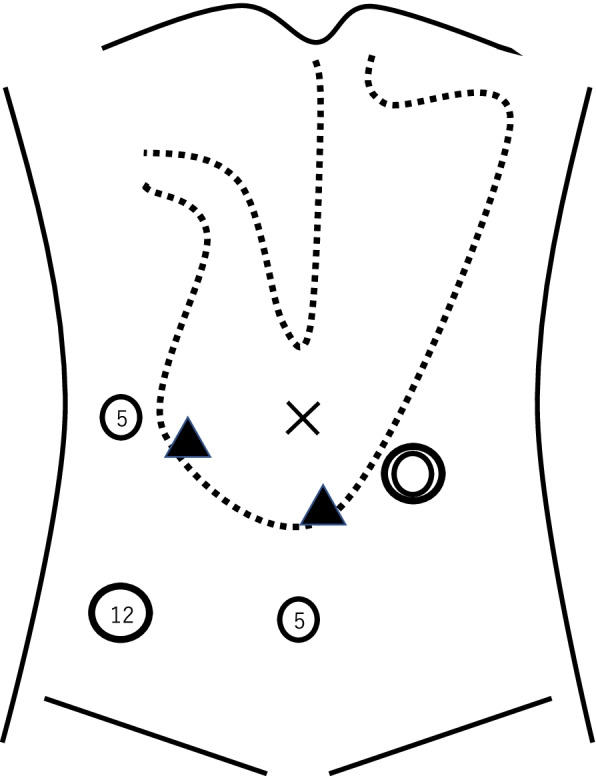
The stomach (a dotted line) was fixed to the abdominal wall. Two black triangles point to the suture sites. A double circle, single circles and numbers point to the sigmoid colostomy, the location and size of the trocars, respectively

**Fig. 4:**
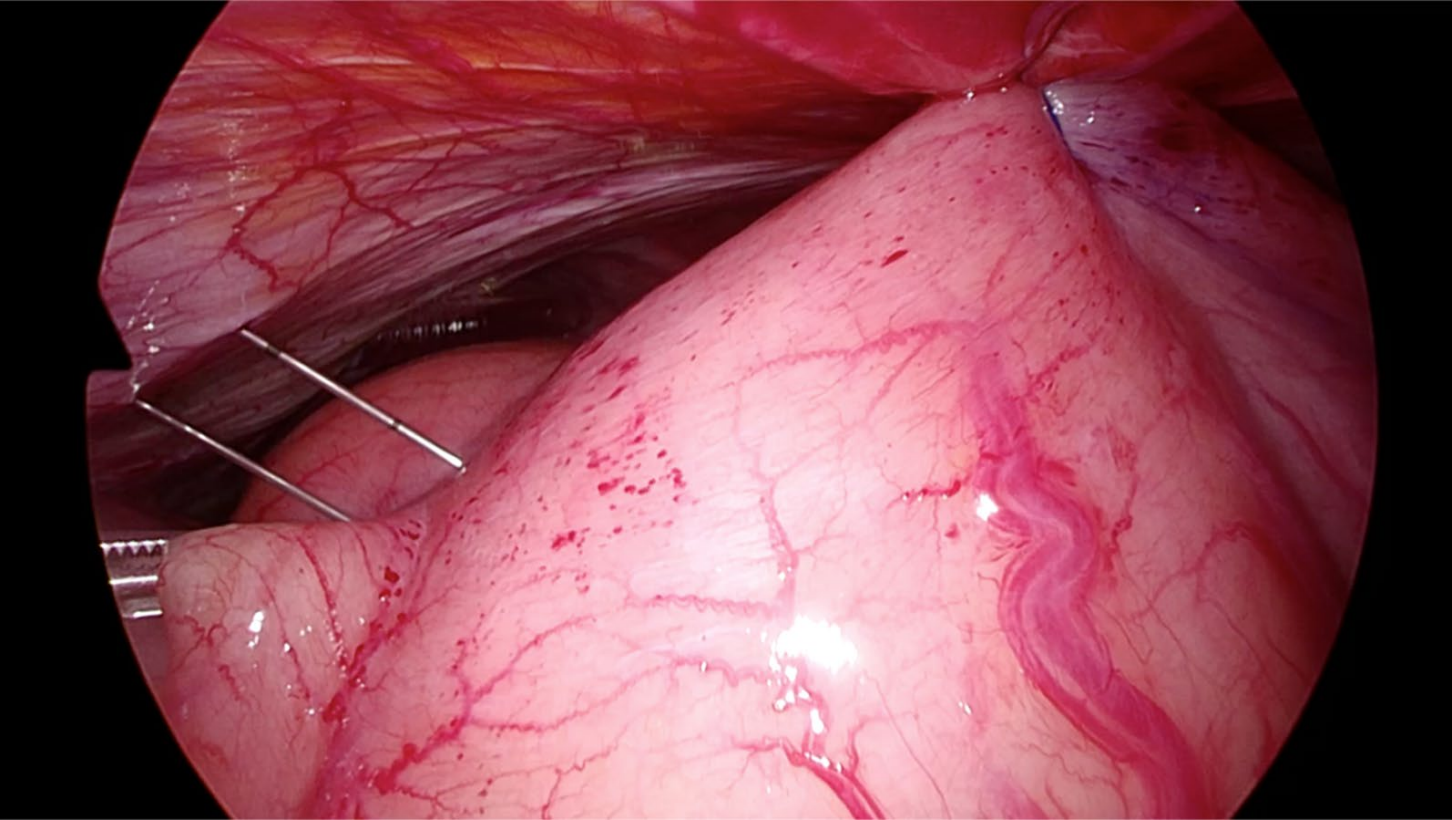
The gastric antrum was fixed to the abdominal wall using a Funada-style loop gastropexy device

**Fig. 5:**
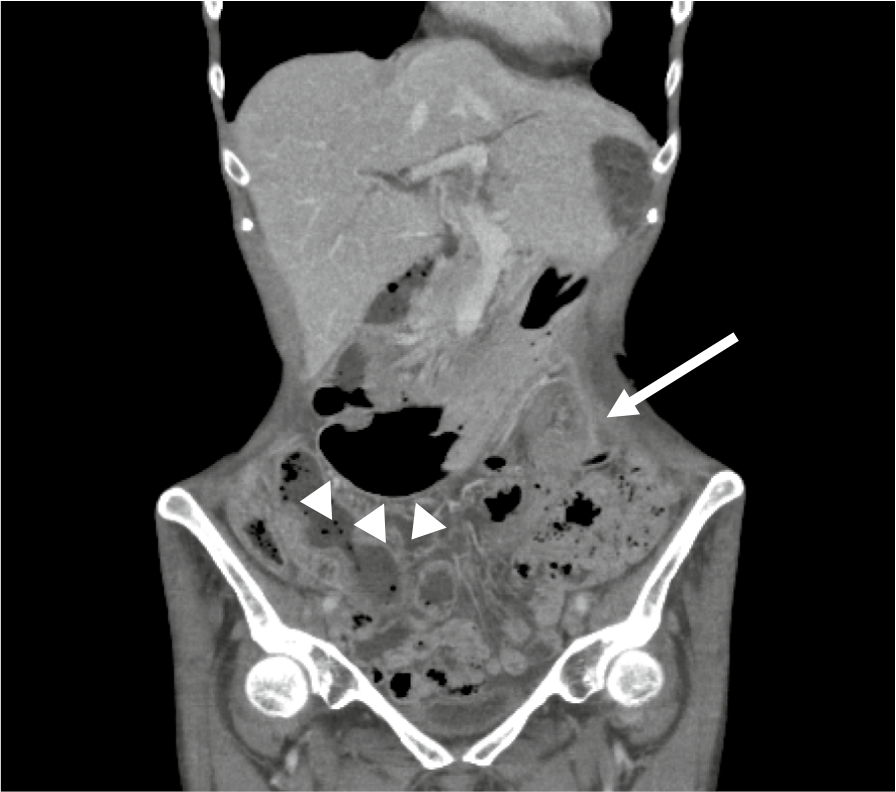
CT showed the position of the stomach after laparoscopic gastropexy was to the right of the lifted sigmoid colon. A white arrow and arrowheads point to the sigmoid colostomy and the stomach, respectively

We inserted trocars of 12 mm, 5 mm, and 5 mm into the right lower abdomen, right lateral abdomen, and mid-lower abdomen, respectively (Fig. 3). The endoscope was inserted into the stomach, and air was passed through it. Two points of the gastric antrum were fixed to the abdominal wall using a Funada-style loop gastropexy device [7] and gastroscopy (Figs. 3 and 4); thereafter, more air was pumped into the stomach. However, the internal hernia did not relapse, and we completed the operation. The amount of blood loss was small, and the operation time was 1 h and 4 min. The postoperative course was uneventful. She started eating the next day and was discharged on postoperative day 5. The internal hernia did not recur until the next surgery. Contrast enhanced CT showed that the pylorus was on the right side of the lifted sigmoid colon (Fig. 5).

A laparoscopic abdominoperineal resection was performed approximately 2 months after the laparoscopic gastropexy. Although it was converted to laparotomy due to the presence of adhesions, the sutures between the stomach and the peritoneum could be removed easily. The internal hernia was treated by suturing the sigmoid colon to the lateral abdominal wall. The postoperative course was good and the internal hernia of the stomach did not recur.

## Discussion

Most postoperative internal hernias are caused by mesenteric defects created by gastrointestinal and colorectal surgeries [8]. Mesenteric defects often lead to intestinal obstruction and rarely to internal hernias of the stomach. Two cases of internal hernias of the stomach following a sigmoid colostomy have been reported; one was treated by suturing the sigmoid stoma to the abdominal wall [3], while the other was treated by changing the intraperitoneal route to an extraperitoneal one [4]. In both cases, an abdominoperineal resection was performed and an end stoma was created. Contrastingly, in the present case, a colostomy with double orifices was created after rectal perforation.

Gastropexy was originally performed for gastric volvulus [9]. Currently, it is often performed laparoscopically [5]. The site and number of the sutures have not been standardized, however, previous reports revealed that laparoscopic gastropexy had good postoperative results [5, 6] and it was adopted in the present case. We have described the case of a 67-year-old patient who underwent laparoscopic gastropexy for an internal hernia of the stomach after sigmoid colostomy.

**Fig. 6:**
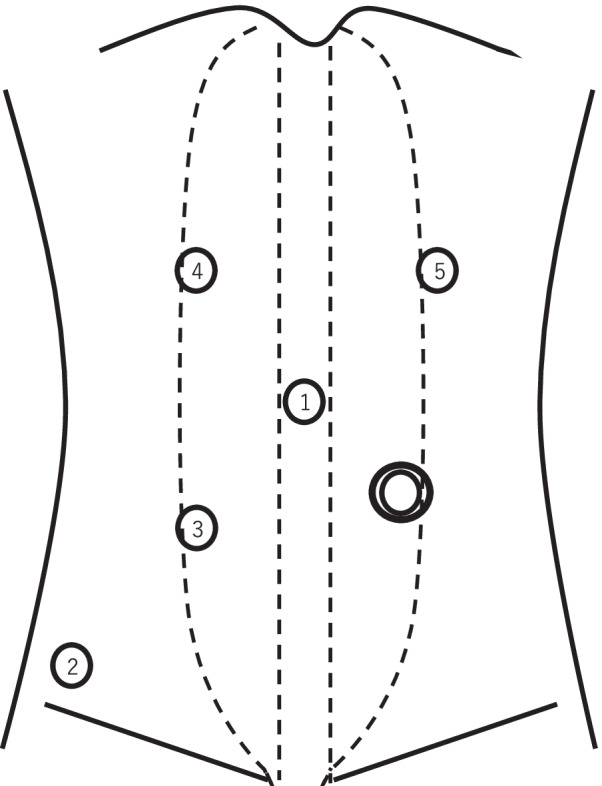
In robotic surgery, the third and fourth trocars penetrate the right rectus abdominis muscle. A dotted line, numbers and double circles indicate the rectus abdominis muscle, order of port insertion and the sigmoid colostomy, respectively

Two previous cases reported that a reliable treatment for hernia was to close the aperture surgically [3, 4]. It was thought best to perform abdominoperineal resection and the aperture closure surgery at the same time, however, since the patient was undergoing chemoradiation therapy and it was difficult to change the date of lengthy surgery, we decided to perform them separately. The next operation was planned to be either laparoscopic or robotic, and the right buttock was partially resected to prevent local recurrence due to rectal perforation. The right rectus abdominis muscle could be used as a reconstructive organ for the defect. However, in robot-assisted surgery, the trocars must be inserted at or near the muscle; therefore, to avoid muscle damage, we chose laparoscopic surgery (Fig. 6).

**Fig. 7:**
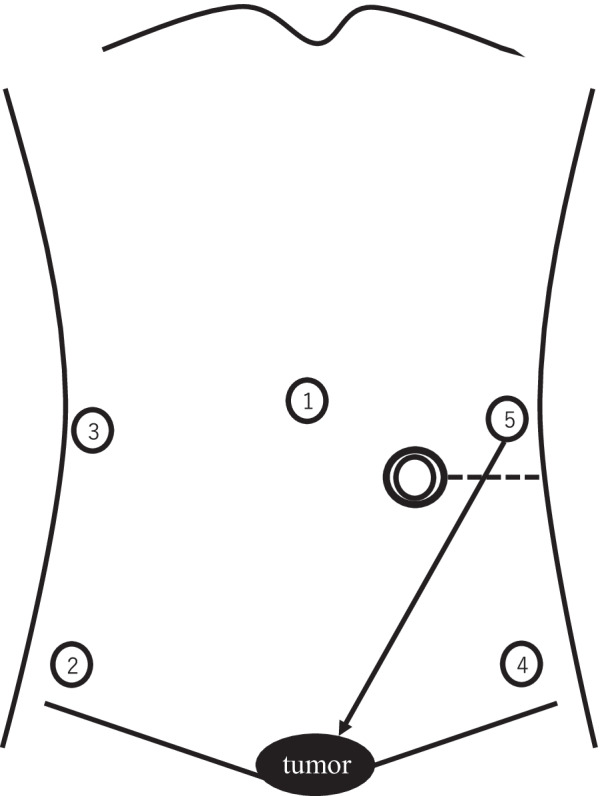
Suturing of the lifted sigmoid colon to the lateral abdominal wall could prevent the movement of the assistant’s forceps from the fifth trocar during the next operation. The dotted line, a black arrow and numbers point to the sigmoid mesentery, direction of forceps and order of port insertion, respectively

Laparoscopic suturing of the lifted sigmoid colon to the lateral abdominal wall was complicated and could prevent the movement of the assistant’s forceps during the next operation (Fig. 7). Changing the intraperitoneal route to an extraperitoneal one was also considered complicated and required cutting the anal side of the stoma. We performed a simple suture fixation of the stomach to the anterior abdominal wall, because the procedure was easy, short, and comprised only two suture fixations. Moreover, the sutures could be removed easily.

In the present case, laparoscopic gastropexy was performed using a Funada-style loop gastropexy device during gastroscopy because we thought that suturing was difficult due to the proximity between the port insertion site and the suture site (Fig. 3). Inflating the stomach during gastroscopy helped confirm an adequate resolution of the hernia and rule out a recurrence. Considering that the preoperative gastroscopy showed gastroptosis, the procedure was considered appropriate.

Because the stomach must be fixed to the right lower abdomen, the location of the right inferior epigastric artery must be confirmed to avoid injuries. Additionally, two fixation points may cause new internal hernias. In the present case, two-point fixation did not cause new internal hernias. Two-point fixation was chosen because one-point fixation could cause stomach volvulus [6]. We believe that laparoscopic gastropexy for internal hernias of the stomach is a good alternative for patients who require additional surgery.

Laparoscopic gastropexy, though simple and minimally invasive, is an incomplete procedure. The most reliable treatment for hernia is to close the aperture surgically. In the present case, we performed laparoscopic gastropexy as the subsequent surgery, and the patient recovered without recurrence. Because this report only details one case, further studies are needed with more cases in the future.

## Conclusions

To our knowledge, this is the first report on the performance of laparoscopic gastropexy in the management of an internal hernia of the stomach after a colostomy. Our observations suggest that laparoscopic gastropexy may be useful for such hernias.

## Data Availability

Not applicable.

## Abbreviation

CT: Computed tomography
